# Comprehensive proteomic analysis of bovine spermatozoa of varying fertility rates and identification of biomarkers associated with fertility

**DOI:** 10.1186/1752-0509-2-19

**Published:** 2008-02-22

**Authors:** Divyaswetha Peddinti, Bindu Nanduri, Abdullah Kaya, Jean M Feugang, Shane C Burgess, Erdogan Memili

**Affiliations:** 1 Department of Basic Sciences, Mississippi State University, Mississippi State, MS 39762, USA; 2 Alta Genetics, Inc., Watertown, WI, USA; 3 Department of Animal and Dairy Sciences, Mississippi State University, Mississippi State, MS 39762, USA; 4 Institute for Digital Biology, Mississippi State University, Mississippi State, MS 39762, USA; 5 Mississippi Agricultural and Forestry Experimental station, Mississippi State, MS 39762, USA

## Abstract

**Background:**

Male infertility is a major problem for mammalian reproduction. However, molecular details including the underlying mechanisms of male fertility are still not known. A thorough understanding of these mechanisms is essential for obtaining consistently high reproductive efficiency and to ensure lower cost and time-loss by breeder.

**Results:**

Using high and low fertility bull spermatozoa, here we employed differential detergent fractionation multidimensional protein identification technology (DDF-Mud PIT) and identified 125 putative biomarkers of fertility. We next used quantitative Systems Biology modeling and canonical protein interaction pathways and networks to show that high fertility spermatozoa differ from low fertility spermatozoa in four main ways. Compared to sperm from low fertility bulls, sperm from high fertility bulls have higher expression of proteins involved in: energy metabolism, cell communication, spermatogenesis, and cell motility. Our data also suggests a hypothesis that low fertility sperm DNA integrity may be compromised because cell cycle: G_2_/M DNA damage checkpoint regulation was most significant signaling pathway identified in low fertility spermatozoa.

**Conclusion:**

This is the first comprehensive description of the bovine spermatozoa proteome. Comparative proteomic analysis of high fertility and low fertility bulls, in the context of protein interaction networks identified putative molecular markers associated with high fertility phenotype.

## Background

Male infertility is a major problem for mammalian reproduction. The nature of sub-fertility due to the male is as complex as that of the female [1]. Infertility due to male factor contributes approximately 40% of the infertility cases in humans. For this reason it is very important to investigate the factors that affect male fertility. Here we used bovine spermatozoa to model human male fertility because cattle provide several advantages as a model for male factor infertility. These include good breeding records fertility data records and progeny records. In cattle breeding, Artificial insemination (AI), a common breeding technique, utilizes semen from genetically superior sires to inseminate cows. In the United States more than ~70% of cows are bred by AI but only ~50% of these matings result in successful full term pregnancy [2]. The underlying molecular events/mechanisms that determine the fertilizing potential of a semen sample are not well defined. A thorough understanding of these mechanisms is essential for obtaining consistently high reproductive efficiency and to ensure lower cost and time-loss by breeder.

Fertility traits of semen can be categorized as compensable or uncompensable [1,3-7]. Defects in compensable traits (motility and morphology) can be overcome by increasing the number of spermatozoa per insemination [1]. Defects in uncompensable traits affect the function of spermatozoa during the later stages of fertilization and in embryonic development [1,8] and as such cannot be compensated. Uncompensable traits include nuclear vacuoles [9], morphological deficiencies that do not suppress movement [4], defective chromatin structure [10]. Low fertility in bulls has an uncompensable component that includes reduced cleavage rate and delayed pronuclear formation following in vitro fertilization [1,11]. Currently available fertility assays assess the defects that affect functional competence of spermatozoa (i.e. capacitation, acrosome reaction, sperm-oocyte interaction) [8,12], however these cannot definitively predict fertility. At present, the molecular nature of sperm fertility defects or biomarkers for accurate fertility prediction is not known [13].

Spermatozoa are transcriptionally inactive so the only comprehensive method to understand the molecular functions in spermatozoa is via proteomics [13]. Published proteomic studies with bull spermatozoa described the sub-proteome of the sperm and functions of proteins from its surrounding cells. Accessory gland (AG) proteins were shown to modulate important sperm functions after ejaculation and in the female reproductive tract such as capacitation, acrosome reaction, sperm-oocyte interaction, and sperm protection [14]. It is known that fertile associated antigen (FAA), a heparin binding protein from seminal vesicles and prostate glands, binds to spermatozoa membrane and modulates heparin-sperm interactions that are indicative of fertility [15]. Two seminal plasma proteins such as, prostaglandin-D-synthetase and osteoponin were more abundant in the semen of high fertility bulls when compared to low fertility bulls [16,17].

Here we describe a comprehensive proteomic analysis of bull sperm using differential detergent fractionation (DDF) two-dimensional liquid chromatography followed by electrospray ionization tandem mass spectrometry (DDF 2-LC ESI MS^2^; [18]). We compared protein expression profiles of sperm from high and low fertility bulls to characterize the differences in fertility at the protein level. Our results show that expression of 2051 and 2281 proteins was specific to high and low fertility bull spermatozoa, respectively and 1518 proteins were common to both. Differential expression of 125 proteins was significant between high and low fertility bull spermatozoa and these proteins are potential biomarkers for bovine male fertility. Biological systems utilize highly complex, interrelated metabolic and signaling pathways to function. Therefore, to identify signaling pathways involved in fertility, we carried out systems modeling of our proteomic datasets using Gene Ontology (GO) and Ingenuity Pathway Analysis (IPA). We identified differences in the signaling pathways between high and low fertility bull spermatozoa and found that EGF and PDGF signaling pathways were specific to high fertility.

## Results

### Proteome profiles of spermatozoa from high and low fertility bulls

**Figure 1:**
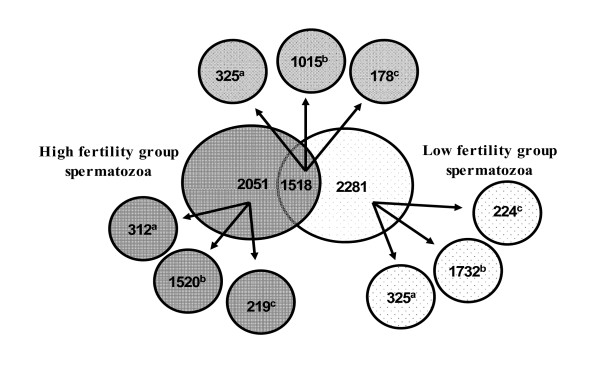
**Comparison of proteins identified in high fertility and low fertility spermatozoa.** Distribution of predicted, known and hypothetical proteins is shown. ^a ^known proteins, ^b ^predicted proteins, ^c ^hypothetical proteins.

We identified 3569 and 3799 proteins in high and low fertility group spermatozoa respectively (see additional file 1). Among these 1518 (20.4%) were common to both groups and 2051 and 2281 proteins were unique to high and low fertility groups respectively (Figure 1). Only those proteins identified by at least three peptides were included in the analysis for differential expression and we identified 125 proteins as differentially-expressed between the high and low fertility spermatozoa. Compared to low fertility bull spermatozoa, expression of 74 proteins increased and there was a decrease in the expression of 51 proteins in high fertility spermatozoa (Table 1). Only a small proportion of proteins identified in this study have been previously described (15.1% of the high fertility group specific and 14.3% of the low fertility group specific proteins (Figure 1)). The majority of the identified proteins are 'predicted' (i.e. predicted based on sequence similarity to known proteins in other species and are frequently found in NRPD database for species that have had their genomes sequenced [19]). We contributed to the annotation of the newly sequenced bovine genome by experimentally confirming the in vivo expression of 4,313 electronically predicted proteins (see additional file 1). We also identified 10.6% and 9.8% 'hypothetical' (i.e. proteins predicted from nucleic acid sequences and that have not been shown to exist by experimental protein chemical evidence [20]) proteins specific to high fertility and low fertility spermatozoa respectively.

**Table 1:** Differentially expressed proteins.

**Accesion**	**Name**	**Peptides**		**∑Xcorr**		**P-value**	**Regulation**
				
		**HF**	**LF**	**HF**	**LF**		
115496714	Actin-like 7B	16	7	60.06	42.11	0.02243	up
77736067	Acyl-CoA thioesterase 9	14	4	39.23	16.48	2.11E-04	up
41386786	A-kinase anchor protein 4	679	581	2581.8	2424.3	0.001694	up
30794280	Albumin	7	1	27.09	5.24	1.49E-04	up
60302887	Aldose reductase	1	3	3.03	12.82	0.03293	down
27807289	Annexin A2	4	10	29.17	26.46	0.04155	down
84490369	ATP synthase, H+ transporting, mitochondrial	16	8	48.01	31.88	0.0333	up
28603752	ATP synthase, H+ transporting, mitochondrial F0	7	1	18.99	7.14	0.005907	up
28461221	ATP synthase, H+ transporting, mitochondrial F1 complex	201	174	765.55	700.71	0.006727	up
28461251	ATPase inhibitory factor 1 precursor	18	10	45.95	30.03	0.01581	up
27807145	Casein kinase 2, alpha prime polypeptide	3	0	11.25	0	5.01E-04	up
60101831	Cytochrome c oxidase subunit III	5	4	17.45	11.47	0.01008	up
84000107	Glycoprotein (transmembrane) nmb	0	3	6.9	8.98	0.0133	down
84000035	Hypothetical protein LOC504736	3	1	8.66	2.04	0.03204	up
115497288	Hypothetical protein LOC506544	4	7	13.54	18.39	0.04931	down
78369248	Hypothetical protein LOC509274	18	10	77.94	51.72	0.01217	up
115496338	Hypothetical protein LOC516024	27	22	109.76	67.93	1.54E-05	up
115495377	Hypothetical protein LOC520260	44	58	201.9	232.07	0.04396	down
114052468	Hypothetical protein LOC532785	8	19	32.19	55.25	0.003313	down
115496742	Hypothetical protein LOC534599	27	11	88.93	61.57	0.02739	up
84000301	Hypothetical protein LOC534927	9	3	32.16	14.67	0.01593	up
115495951	Hypothetical protein LOC540767	20	12	62.88	41.8	0.004421	up
94966950	Hypothetical protein LOC614199	4	0	10.84	1.66	0.007311	up
84000391	Hypothetical protein LOC615316	11	5	35.17	24.34	0.02524	up
115497750	Hypothetical protein LOC617117	19	12	58.06	44.14	0.03432	up
116004271	Hypothetical protein LOC767959	4	0	12.9	3.03	0.04429	up
27805989	Lysyl oxidase-like 4	1	3	1.59	7.16	0.03124	down
27806307	Mitochondrial ATP synthase, O subunit	13	8	51.79	34.78	0.03731	up
28461275	NADH dehydrogenase (ubiquinone) 1 alpha subcomplex	13	3	48.9	27.96	0.05046	up
28461255	NADH dehydrogenase (ubiquinone) 1 beta subcomplex	2	4	3.67	13.56	0.01051	down
62751972	Potassium voltage-gated channel shaker-related	3	0	5.86	0	1.21E-04	up
119891540	PREDICTED: glutathione S-transferase kappa 1	3	3	10.97	8.47	0.01147	up
119887606	PREDICTED: hypothetical protein	1	3	2.03	8.02	0.04382	down
119903031	PREDICTED: hypothetical protein	5	15	25.57	46.02	2.23E-04	down
119908822	PREDICTED: hypothetical protein	3	1	7.23	1.65	0.03051	up
119888977	PREDICTED: hypothetical protein	11	3	37.09	22.02	0.03232	up
119905186	PREDICTED: hypothetical protein	0	3	0	6.3	1.90E-05	down
76661674	PREDICTED: hypothetical protein	1	4	2.22	9.87	0.01595	down
119901076	PREDICTED: hypothetical protein	1	5	9.84	11.22	0.04157	down
119918378	PREDICTED: hypothetical protein	3	15	16.31	36.68	5.24E-04	down
61843441	PREDICTED: hypothetical protein	0	4	0	9.99	4.37E-05	down
119901737	PREDICTED: hypothetical protein	4	6	20.4	18.24	0.02245	down
119904572	PREDICTED: hypothetical protein	3	0	6.15	4.95	0.01124	up
119884876	PREDICTED: hypothetical protein	3	2	9.65	2.72	0.03549	up
76631114	PREDICTED: hypothetical protein	35	15	106.7	81.25	0.02224	up
119923822	PREDICTED: hypothetical protein	63	51	205.06	170.94	0.001919	up
76644873	PREDICTED: hypothetical protein isoform 2	3	0	7.78	1.83	0.03381	up
76645752	PREDICTED: hypothetical protein isoform 4	1	4	1.62	9.68	0.003268	down
119912558	PREDICTED: hypothetical protein isoform 6	4	0	10.87	0	7.37E-12	up
119893872	PREDICTED: hypothetical protein LOC535130	5	1	14.93	12.61	0.03256	up
76687954	PREDICTED: hypothetical protein, partial	3	0	7.7	1.95	0.04264	up
119925886	PREDICTED: hypothetical protein, partial	3	0	6.76	5.69	0.01623	up
119895251	PREDICTED: profilin 3	2	4	12.89	19.46	0.01716	down
119922439	PREDICTED: similar to 1700016M24Rik protein	3	0	8.17	0	1.04E-04	up
119879571	PREDICTED: similar to AAT1-alpha	22	12	69.77	50.74	0.01412	up
119912554	PREDICTED: similar to Ace protein	15	4	55.74	20.29	8.69E-06	up
61878077	PREDICTED: similar to Actin-related protein T1	7	2	16.82	7.44	0.02775	up
119928361	PREDICTED: similar to ADAM metallopeptidase	7	2	23.85	10.3	0.01155	up
119913547	PREDICTED: similar to ADAM metallopeptidase with thrombospondin type 1 motif, 17 preproprotein	5	0	12.58	0	1.24E-07	up
119903267	PREDICTED: similar to ALMS1 protein	4	0	10.2	2.89	0.02294	up
119892487	PREDICTED: similar to Ankyrin repeat domain-containing	1	3	1.88	7.82	0.03473	down
76657564	PREDICTED: similar to calmodulin	10	5	23.53	16.83	0.04331	up
119901005	PREDICTED: similar to centrosomal protein 110kD	3	0	8.38	2.06	0.03921	up
119893858	PREDICTED: similar to chromosome 13 open reading	0	3	0	6.84	4.35E-04	down
61814552	PREDICTED: similar to Cytochrome c oxidase subunit	13	10	41.99	47.17	0.01639	up
119904416	PREDICTED: similar to diaphanous homolog 3	0	3	0	7.64	3.85E-04	down
119915202	PREDICTED: similar to DNAH8, partial	12	3	33.72	16.39	0.001961	up
119927503	PREDICTED: similar to DNAH8, partial	6	1	22.2	8.36	0.04519	up
119911633	PREDICTED: similar to EF-hand calcium binding domain 5	0	3	0	7.52	3.23E-05	down
119888835	PREDICTED: similar to EPH receptor A8	4	0	9.46	1.9	0.009866	up
119895747	PREDICTED: similar to FAT tumor suppressor 2	12	3	28.56	32.84	1.15E-04	up
119919673	PREDICTED: similar to ferritin L subunit isoform	1	4	0.57	10.42	3.74E-05	down
119909426	PREDICTED: similar to fertilin alpha	12	27	63.45	89.9	0.003791	down
119919953	PREDICTED: similar to filamin	0	3	0	6.94	4.54E-04	down
76662361	PREDICTED: similar to GFHL3075 isoform 3	3	0	8.3	7.63	0.01657	up
61820991	PREDICTED: similar to GK2 protein	16	9	54.86	31.79	0.01383	up
119901324	PREDICTED: similar to HECT domain and ankyrin repeat containing, E3 ubiquitin protein ligase 1 isoform 1	2	3	6.09	6.74	0.01418	down
119895512	PREDICTED: similar to HIST1H4I protein	3	0	6.36	1.06	0.007699	up
119915532	PREDICTED: similar to histone H2b-616	4	10	17.41	34.08	0.005828	down
76613952	PREDICTED: similar to histone H4	2	2	7.48	5.88	0.02561	down
76642199	PREDICTED: similar to Izumo sperm-egg fusion 1	39	24	133.67	99.29	0.005314	up
119890207	PREDICTED: similar to KIAA0191	18	26	70.99	64.29	0.01024	down
119891377	PREDICTED: similar to KIAA0225 isoform 1	1	4	1.01	9.43	5.50E-04	down
119890395	PREDICTED: similar to KIAA0467 protein	0	3	0	7.91	3.74E-06	down
119902048	PREDICTED: similar to KIAA1305 protein	0	3	0	7.91	0.001712	down
119906772	PREDICTED: similar to KIAA1429 protein isoform	0	3	0	6.98	4.55E-04	down
119912552	PREDICTED: similar to KIAA1636 protein	3	0	7.44	0	1.53E-04	up
119891313	PREDICTED: similar to KIAA1793 protein	12	8	52.91	37.11	0.0228	up
119909205	PREDICTED: similar to KIAA2017 protein isoform	6	0	17.51	6.69	0.01123	up
76664109	PREDICTED: similar to LOC505732 protein	4	6	14.08	12.97	0.01832	down
76641602	PREDICTED: similar to LOC507431 protein isoform	10	7	31.93	36.41	0.03413	up
119902010	PREDICTED: similar to LOC512571 protein	47	34	189.9	155.47	0.03649	up
119905900	PREDICTED: similar to NDRG3	0	3	0	7.3	1.80E-04	down
76612380	PREDICTED: similar to nestin	0	3	0	6.85	1.25E-04	down
119911939	PREDICTED: similar to netrin-1	2	4	2.84	8.74	0.01328	down
119894490	PREDICTED: similar to obscurin, cytoskeletal calmodulin and titin-interacting RhoGEF, partial	1	3	1.45	7.16	0.03376	down
119905455	PREDICTED: similar to Pitrilysin metallopeptidase	1	6	2.49	22.6	3.23E-05	down
76618065	PREDICTED: similar to Pou6f1 protein	4	1	15.92	11.31	0.006931	up
119894859	PREDICTED: similar to Protein KIAA1543 isoform	1	3	1.36	8.01	0.008488	down
119893105	PREDICTED: similar to protein kinase A binding protein	135	113	480.21	392.89	2.35E-05	up
76627105	PREDICTED: similar to RAB2B, member RAS oncogene	7	16	29.46	52.58	0.004367	down
119912290	PREDICTED: similar to RIKEN cDNA 4121402D02	3	0	7.29	0	2.00E-07	up
119914167	PREDICTED: similar to RIKEN cDNA A530050D06 gene	6	8	12.57	21.82	0.007372	down
119903563	PREDICTED: similar to RNA polymerase I polypept	3	6	7.15	15.59	0.03155	down
119910233	PREDICTED: similar to sca1	3	0	7.43	1.65	0.0301	up
119916698	PREDICTED: similar to Septin 12	0	3	0	8.2	2.26E-07	down
119903556	PREDICTED: similar to sulfotransferase K1	0	3	0	6.79	1.49E-04	down
119902145	PREDICTED: similar to telomerase-associated protein	3	0	8.27	0	8.45E-06	up
119914302	PREDICTED: similar to trans-Golgi p230	4	0	8.82	2.85	0.01983	up
119917225	PREDICTED: similar to TRRAP protein	2	5	2.92	11.86	0.002132	down
119917582	PREDICTED: similar to TUBA	3	1	9.42	3.39	0.03158	up
119912117	PREDICTED: similar to Tumor necrosis factor receptor	7	0	17.77	0	6.66E-12	up
119903686	PREDICTED: similar to ubiquitin specific protease 34 isoform1	1	4	2.31	9.99	0.01666	down
77736091	Prohibitin	7	3	22.59	9.08	0.03313	up
114052901	Rhabdoid tumor deletion region gene 1	10	2	31.71	15.41	0.009593	up
84000339	Sperm associated antigen 6	4	1	13.76	2.2	0.02495	up
87196516	Sperm mitochondria-associated cysteine-rich protein	3	0	9.82	3.51	0.03455	up
115495195	Tektin 1	27	19	107.5	76.3	0.0155	up
84000201	Transmembrane protein 5	3	1	6.27	6.97	0.004588	up
61888856	Triosephosphate isomerase	40	26	158.4	122.55	0.005981	up
27807143	Ubiquinol-cytochrome c reductase core protein II	7	2	33.24	16.14	0.02472	up

List of differentially expressed proteins in high fertility (HF) group spermatozoa when compared to low fertility group. (LF) spermatozoa In this table we provided the information about number of peptides, Sequest cross correlation (∑Xcorr) score and P value for each protein in high and low fertility group spermatozoa respectively.

Predicted and hypothetical proteins do not have any functional annotation associated with them and they represent ~80% of differentially expressed proteins between high and low fertility spermatozoa (Table 1). This poses a problem for meaningful biological modeling of our data without carrying out some functional annotation first. Therefore, we annotated all differentially expressed proteins in our data sets using AgBase GO resources.

### Membrane and nuclear proteins

Membrane and nuclear proteins are fundamental for inter and intra cellular signaling and are thus fundamental for modeling cell-cell interactions. Sperm oocyte fusion is a key element for fertilization. This process is facilitated by sperm surface proteins and leads to specific binding of the sperm surface-active component with the egg zona pellucida and, ultimately, sperm-egg fusion [21]. To identify proteins from the sperm membrane and the nucleus which function in cell fusion, we focused on membrane and nuclear proteins identified in our datasets. Based on the GO associations of known proteins, 40.6% (395) are membrane proteins. We also identified 112 nuclear proteins based on GO associations. Biological process annotation of membrane proteins revealed that majority of membrane proteins involved in transport (33%), cell communication (18%) and metabolism (17%).

**Figure 2:**
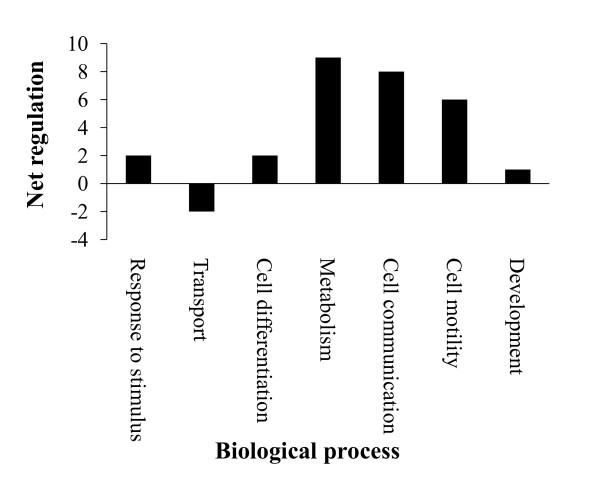
**Overall effects in GO Slims of differentially expressed proteins of high and low fertility spermatozoa.** Biological process GO annotations of all significantly altered proteins between high and low fertility spermatozoa were used to generate GO Slims. For each GO Slim, the difference in the numbers of proteins with increased expression and the number of proteins with decreased expression (relative to low fertility spermatozoa) was calculated to estimate the net regulatory effect.

We GO annotated all differentially expressed proteins and applied the generic GO Slim [22] to identify 7 functional super-categories represented in differentially expressed proteins in high fertility spermatozoa. Most GO Slim categories, including processes such as metabolism, cell communication and cell motility showed overall up regulation of protein expression in the high fertility group while transport proteins showed an overall down regulation in the high fertility group (Figure 2).

### High fertility and low fertility sperm proteomes: molecular network and pathway analysis

Protein identification from biological samples on a global scale is important. However, there is a need to move beyond this level of analysis; Instead of simply enumerating a list of proteins, the analysis needs to include their interactions as parts of complexes, pathways and biological networks. To achieve this level of analysis with our high fertility and low fertility spermatozoa proteomic datasets we used Ingenuity Pathway Analysis (IPA). At IPA thresholds for significance, 71, and 73 networks and 68, and 73 functions/diseases were significantly represented in the proteomes of high fertility and low fertility spermatozoa respectively. The top 10 functions/diseases (ranked based on significance), and the associated signaling pathways are shown in Table 2 and Table 3 for proteomes of high and low fertility groups respectively. Analysis of the top 10 functions revealed that functions like cellular movement, cell to cell signaling and interaction were identified only in the high fertility sperm proteome (Table 2). Whereas, functions like cell death and reproductive system disease were identified only in the low fertility sperm proteome (Table 3).

**Table 2:** Top ten functions/diseases and their respective top ten signaling pathways in high fertility group spermatozoa.

	**Signaling Pathways**									
	
**Functions & diseases**	EGF signaling	PDGF signaling	Integrin signaling	Amyloid processing	Complement and Coagulation cascade	PPAR signaling	Neurotrophin signaling	Huntingtons disease signaling	IGF1 Signaling	Apoptosis signaling
Cell cycle	12	14	13	10	4	11	11	13	14	10
Cellular movement	9	12	25	10	11	13	9	12	11	14
Connective tissue development and function	3	4	7	5	2	2	3	2	5	2
Cellular assembly and Organization	3	5	15	9	3	4	4	5	4	7
Cell morphology	15	17	31	10	6	10	15	16	14	12
Cardio-vascular disease	4	5	3	5	3	2	3	7	3	7
Lipid metabolism	2	2	2	2	1	2	2	3	2	2
Small molecule Biochemistry	9	10	12	12	1	6	8	9	12	12
Cell to cell signaling and interaction	6	8	22	3	13	3	5	6	7	7
Post translational modification	8	10	14	13	1	7	7	9	9	10

EGF: Epidermal Growth Factor; PDGF: Platelet Derived Growth Factor; IGF1: Insulin Growth Factor-1.

**Table 3:** Top ten functions/diseases and their respective top ten signaling pathways in low fertility group spermatozoa.

	**Signaling Pathways**									
	
**Functions & Diseases**	Cell cycle:G2/M DN Adamage check point regulation	Integrin signaling	Apoptosis signaling	MAPK signaling	Amyloid Processing	VEGF signaling	G-protein coupled receptor signaling	PTEN signaling	Actin Cyto-skeleton signaling	Axonal guidance signaling
1.Cell cycle	10	11	11	10	5	8	8	10	10	7
2.Cell morphology	3	26	13	18	7	11	18	14	31	26
3.Post translational modification	9	12	9	11	9	5	11	9	14	11
4.Cellular assembly and Organization	3	18	4	10	5	5	7	4	23	20
5.Lipid metabolism	0	2	3	5	5	3	5	2	2	4
6.Small molecule biochemistry	5	9	10	11	8	4	13	7	11	11
7.Connective tissue disorder	1	12	1	11	2	4	10	6	11	1
8.Gene Expression	8	6	8	16	6	5	16	11	6	8
9.Cell death	7	17	14	17	8	8	14	13	15	18
10.Reproductive system disease	9	5	6	9	2	7	4	8	14	12

MAPK: Mitogen Activated Protein Kinase; VEGF: Vascular Endothelial Growth Factor; PTEN: Phosphatase and tensin homolog deleted on chromosome ten.

**Figure 3:**
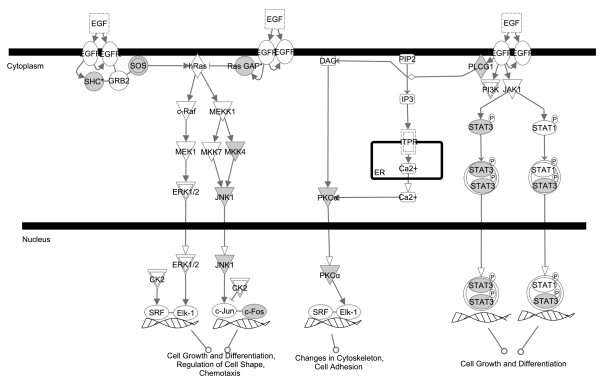
**EGF signaling pathway generated by the Ingenuity Pathway Analysis (IPA) software.** EGF and PDGF signaling pathways were the top two pathways in the top 10 functions/diseases associated with the high fertility spermatozoa (Table 2). Each node represents a protein; proteins in shaded nodes were found in the high fertility spermatozoa dataset (see additional file 1) while proteins in clear nodes were not found in the high fertility spermatozoa dataset.

Compared to low fertility sperm proteome (9), the high fertility sperm proteome (20) had a 2-fold enrichment in signaling pathways. However, the number of significant metabolic pathways represented was comparable between the low (8) and high (9) fertility spermatozoa. Epidermal growth factor (EGF) signaling was the most prominent signaling pathway specific to high fertility sperm (Figure 3). EGF signaling is known to promote proliferation, survival, and differentiation of a wide variety of mammalian cells [23]. In addition to the EGF signaling pathway, platelet derived growth factor (PDGF) signaling, peroxisome proliferated activator receptor (PPAR) signaling, interleukin(IL) -4 signaling, NF-kβ signaling, chemokine signaling, and insulin growth factor (IGF)-1 signaling were identified only in high fertility spermatozoa. In low the fertility group, Cell cycle: G2/M DNA damage check point regulation was the most significant pathway followed by integrin signaling.

### Proteins with significantly altered expression: molecular network and pathway analysis

Systems analysis of global proteomes revealed that some signaling pathways are differentially represented between the high and low fertility group spermatozoa. To further analyze these differentially expressed pathways, we carried out IPA analysis with just the 125 differentially expressed proteins. In high fertility spermatozoa, expression of 74 proteins was increased when compared to low fertility spermatozoa. IPA analysis identified three significant networks with scores of 22, 19, and 13 respectively. Proteins identified in the top three networks are participants in EGF signaling, PDGF signaling, oxidative phosophorylation, and pyruvate metabolism pathways. Expression of two proteins, ATP synthase, H+ transporting, mitochondrial F1 complex (ATP5B), and cytochrome c oxidase subunit III (COX3) involved in oxidative phosphorylation and casein kinase II involved in EGF signaling and PDGF signaling were higher in the high fertility spermatozoa compared to low fertility spermatozoa (Table 1). IPA also identified pyruvate metabolism as the most significant pathway in up regulated proteins of high fertility spermatozoa. In the low fertility sperm proteome, expression of 51 proteins increased when compared to high fertility spermatozoa. IPA analysis identified two significant networks in highly expressed proteins of low fertility sperm. Proteins identified in the top two significant networks are participants in integrin signaling and estrogen receptor signaling.

## Discussion

Male fertility can be described as the success by spermatozoa to fertilize oocytes and of the resulting zygotes continue on through embryonic and fetal development until birth [11]. In this study we used bovine spermatozoa to study fertility as it can serve as a model for understanding human male infertility and reproductive diseases. Studying Bovine male fertility on its own merit has implications in agro-economics involving cattle industry worldwide.

A spermatozoon must reach the site of fertilization and be capacitated for successful fertilization to occur. A subsequent step is the acrosome reaction characterized by fusion of a spermatozoon outer acrosomal membrane with overlying plasma membrane [8]. The molecular mechanisms and signal transduction pathways mediating the processes of capacitation and acrosome reaction have been partially defined [8]. Bull sperm cytosolic fraction proteomic analysis showed enrichment for tyrosine kinases which are essential for phosphorylation of specific sperm proteins during capacitation [24]. The abundance of a variety of proteins from cells surrounding the sperm has been proposed to indicate male fertility [2,14,15]. Most of the studies used 2-dimensional electrophoresis (2-DE) for isolation and identification of sperm proteins [13,25-28]. To our knowledge this is the first comprehensive non-electrophoretic proteomic study of bull sperm proteome. The aim of our study was to identify proteins that were differentially expressed between high and low fertility bull spermatozoa and interrelated metabolic and signaling pathways that have a role in fertility.

We identified 125 proteins as differentially expressed in between the high and low fertility sperm even though 1518 proteins were common to both groups and about 2000 were unique to each. The reasons for this apparent discrepancy are that we took a conservative approach to the statistical analysis: only proteins identified by at least three peptides were included in the analysis for differential expression and the statistical method used in *ProtQuant *is very conservative. *ProtQuant *specifically address the issue of "missing" mass spectra that occurs in all 2-D LC MS^2 ^-based expression proteomics methods. No other published method (either non-isotopic or isotopic) addresses this issue. Missing mass spectra are due to the inherent limitations of the mass spectrometers, the probabilistic nature of sampling and the cutoffs used to determine "true" assignments of peptides to mass spectra [29]. *ProtQuant *is highly conservative method which is based on sum of Xcorr method itself increases the specificity of spectral counting and reduce the type I errors of differential expression. Regardless, proteins were analyzed from each of three of the areas represented in Figure 1 and differentially-expressed proteins occurred in all three (i.e. proteins unique to the high and low fertility sperm as well as those common to both).

From proteome profiles of specific cells or tissues, one acquires large datasets that are inherently complex. As a result we consider it beneficial to model our bovine sperm proteome data sets using GO and IPA. From GO associations of differentially expressed proteins we found that there was a comparative up regulation of three biological processes in high fertility spermatozoa: metabolism, cell communication and cell motility (Fig 2).

Up regulation of metabolism is consistent with the fact that capacitation is coupled to a specific type of metabolism, that is glycolysis or oxidative respiration [30]. Pyruvate metabolism and glycolysis were the top most significant metabolic pathways represented in high fertility sperm proteome by IPA. In glycolysis, expression of pyruvate kinase (PKM2) was higher in high fertility spermatozoa. PKM2 catalyzes the production of pyruvate and ATP from phosphoenol pyruvate. Pyruvate formed in this process serves as an energy source for cells [31]. Impaired or lower pyruvate metabolism could limit the cell's ability to produce energy and this could be one of the reasons for reduced fertility in the low fertility group.

Expression of COX 3 and ATP5B involved in oxidative respiration was higher in high fertility spermatozoa compared to low fertility spermatozoa. COX3 is a member of the large transmembrane protein complex found in the mitochondrion and is the last protein in the electron transport chain. Coupling of electron transport to oxidative respiration maintains the high mitochondrial transmembrane potential required for mitochondrial ATP production [32]. ATP5B catalyzes the production of ATP from ADP in the presence of a proton gradient across the mitochondrial membrane and this ATP is utilized for the motility of sperm and capacitation [33].

Communication between sperm and oocyte is critical for successful fertilization. We found that there was up regulation of cell communication in the high fertility sperm proteome when compared to low fertility sperm proteome (Figure 2). To bring about cell to cell communication several signaling pathways are necessary. EGF signaling and PDGF signaling were the top two significant signaling pathways identified in high fertiliy spermatozoa. EGF and PDGF signaling pathways stimulate tyrosine phosphorylation of various MAP kinases and their upstream activators MEK1, MEK2 and MEKK [34,35]. EGF signaling has an important role in sperm capacitation as it stimulates tyrosine phosphorylation of many proteins [36]. In addition, EGF signaling also activates phospholipase C (PLC) [36] (Figure 3). PLC is important for the acrosome reaction (AR), fertilization and embryo development. PLC catalyzes the production of inositol 1, 4, 5-triphosphate (IP3) from phosphatidylinositol 4, 5-biphosphate. IP3 generated by PLC activates the extra cellular calcium influx required for the AR via binding to the IP3 receptor (IP3R) gated calcium channel located on the acrosome membrane [37]. Mutations in mouse PLCB1 reduced the AR rate, fertilization rate and embryo development [38]. EGF signaling was specific to high fertility bull sperm. Defects in EGF signaling in low fertility spermatozoa may prevent capacitation.

Expression of casein kinase 2 (CKII) prime poly peptide in EGF signaling was higher in high fertility spermatozoa compared to low fertility spermatozoa (Table 1). CKII is preferentially expressed in late stages of spermatogeneis and is involved in sperm chromatin decondensation after sperm oocyte fusion [39,40]. CKII deficient mice are infertile with oligospermia and globozoospermia[40]. EGF signaling also induces actin polymerization in bovine sperm capcitation [41]. Actin polymerization is essential for incorporation of sperm into egg cytoplasm [42] and for sperm nuclei decondensation [43].

Comparing the proteome profiles of bull sperm of high and low fertility showed some molecular features associated with low fertility. Cell cycle: G2/M DNA damage check point regulation was the topmost significant signaling pathway followed by integrin signaling in low fertility bull sperm (Table 3). The G2/M DNA damage checkpoint could help in maintaining the integrity of the genome during different stages of development. Progression through different phases of the cell cycle requires the sequential activation of various cyclin dependent kinases and these kinases in turn are regulated by integrin signaling. Integrin signals are necessary for cells to traverse the cell division cycle [44]. These two pathways may be a compensatory response for reproductive system disease function which was identified only in low fertility sperm (Table 3).

In addition to differences in signaling and metabolic pathways between high and low fertility spermatozoa, we identified differences in protein expression that had implications in sperm motility. Expression of A-kinase anchor protein-4 (AKAP4) was significantly higher in high fertility spermatozoa (Table 1). AKAP4 is a major fibrous sheath protein of the principal piece of the sperm flagellum. AKAP4 recruits Protein kinase A to the fibrous sheath and facilitates local phosphorylation to regulate flagellar function in humans [45]. It also serves as a scaffolding protein for signaling proteins and proteins involved in metabolism. Higher expression of AKAP4 in the high fertility group sperm could result in higher motility.

## Conclusion

In summary, this is the first comprehensive description of the spermatozoa proteome of bovine. Comparative proteomic analysis of high fertility and low fertility bulls, in the context of protein interaction networks identified putative molecular markers associated with high fertility phenotype. We observed marked differences in signaling and metabolic pathways between high fertility and low fertility spermatozoa that have implications in sperm capacitation, acrosomal reaction and sperm-oocyte communication.

## Methods

### Selection of high and low fertility bulls

Frozen semen samples and bull fertility data (see additional file 2) from six mature and progeny tested Holstein bulls with satisfactory semen quality were provided by Alta Genetics (Watertown, WI).

#### Sample and Data Sources

The fertility data were established by a progeny testing program named Alta Advantage^®^, which is the industry's most reliable source of fertility information. It consisted of insemination records collected from 180 well managed partner dairy farms located in different geographical regions across the United States. This breeding program provided the advantages of DNA verification of the paternity of the offspring, and diagnosed pregnancies by veterinary palpation, instead of just relying on non-return rates 60–90 days after breeding.

#### Bull Fertility Prediction

To predict fertility of the bulls from the given source, a sub-set of data were generated consisting of 962,135 insemination records from 934 bulls with an average of 1,030 breedings ranging from 300 to 15,194. The environmental and herd management factors that influence fertility performance of sires were adjusted using threshold models which were similar to previously published models by Zwald et al [46,47]. Parameters estimation and fertility prediction were obtained using Probit.F90 software developed by Y. M. Chang [48].

Therefore, for the definition of fertility, instead of relying only on the number of pregnant cows (verified using palpation by a veterinarian or ultrasound examination) divided by the total number of cows examined for pregnancy, we considered the outcome of each breeding event and adjusted the environmental factors such as the effects of herd-year-month, parity, cow, days in milk, sire proven status (young, proven, colored) in order to rank the bulls based on their breeding values for fertility. Further, the fertility of each bull was calculated and expressed as the percent deviation of its conception from the average conception of all bulls having at least 300 breeding in the data set.

#### Selection of high and low fertility bulls

For this study, we used an arbitrary threshold for classifying high and low fertility bulls. However, the bulls scoring highest and lowest fertility deviation from average with highest reliability (>1,000 breeding/bull) were selected for this study. The differences in the average fertility indexes between high and low fertility groups were 5.46% which was obtained from bulls having adequate records for higher reliability. While three bulls which were scored 5.3% above the average were considered high fertile, three bulls which were scored 10.76% below the average were defined as low fertility (see additional file 2). Two separated pools of sperm cells (3 × 10^8^) were constituted by mixing equal amounts of sperm cells from either three low or three high fertility bulls. The experiment was replicated three times.

### Isolation of pure sperm cells

Spermatozoa were collected from high and low fertility bulls and frozen in 0.25 ml straws. For each bull, the total spermatozoa collected were purified by Percoll gradient centrifugation: 90% Percoll solution in water was prepared with DL-Lactate (19 μM), CaCl_2 _(2 μM), NaHCO_3 _(25 mM), MgCl_2 _(400 μM), KCl (3 μM), NaH_2_PO_4 _(310 μM), NaCl (2 mM) and Hepes (10 mM). 90% Percoll solution was diluted to 45% with sperm diluent medium (1 mM pyruvate, 10 mM Hepes, 0.021 mM DL-Lactate in Tyrode's salt solution, pH 7.4). A density gradient of Percoll was prepared in an Eppendorf tube (0.1 ml of 90% fraction under 1 ml of the 45% fraction). Spermatozoa were thawed at 35°C for 1 min and layered on top of the percoll gradient. The spermatozoa were pelleted by centrifugation (956 *g*; 15 min) followed by two washes in phosphate-buffered solution (PBS) (956 *g*; 5 min,). The total sperm count was obtained using an Improved Neubauer Hemacytometer and 10^8 ^sperm cells were aliquoted and stored at -80°C.

### Protein extraction by DDF

DDF sequentially extracts proteins from different cellular compartments using a series of detergents and this off-line pre-fractionation step in sample preparation increases the proteome coverage. Another advantage of using DDF is that based on the DDF fractions from which proteins are identified, proteins can be found in different cellular locations. Proteins were isolated using DDF as previously described [18]. Cytosolic proteins were extracted by six sequential incubations in a buffer containing digitonin (10 min each); next a fraction containing predominantly membrane proteins was isolated by incubating the cells in 10% Triton X-100 buffer for 30 min and then removing the soluble protein. Nuclear DDF buffer containing deoxycholate (DOC) was then added to the remaining insoluble material and subjected to freeze-thawing to disrupt the nucleus. Nuclear proteins were collected from the resulting soluble fraction and the sample was then aspirated through an 18 g needle and treated with a mixture of DNase I (50U, Invitrogen, Carlsbad CA;) and RNase A (50 mg; Sigma-Aldrich, St Louis, MO) at 37°C for 1 h) to digest nucleic acids. Any remaining pellet, containing the least soluble proteins, was treated with a buffer containing 5% SDS.

### Proteomics

Proteomic analysis was carried out with triplicate samples of spermatozoa from the high fertility group and low fertility group spermatozoa as described [19]. Proteins were precipitated with 25% tricholoroacetic acid to remove salts and detergents. Protein pellets were resuspended in 0.1 M ammonium bicarbonate with 5% HPLC grade acetonitrile (ACN), reduced (5 mM, 65°C, 5 min), alkylated (iodoacetamide, 10 mM, 30°C, 30 min) and then trypsin digested until there was no visible pellet (sequencing grade modified trypsin, Promega; 1:50 w/w 37°C, 16 h). Peptides were desalted using a peptide macrotrap (Michrom BioResources, Inc., Auburn, CA) and eluted using a 0.1% trifluoroacetic acid, 95% ACN solution. Desalted peptides were dried in a vacuum centrifuge and resuspended in 20 μL of 0.1% formic acid and 5% ACN. LC analysis was accomplished by strong cation exchange(SCX) followed by reverse phase liquid chromatography (RP-LC) coupled directly in line with an ESI ion trap mass spectrometer (LCQ Deca XP Plus; ThermoElectron Corporation; San Jose, CA). Samples were loaded into a LC gradient ion exchange system (Thermo Separations P4000 quaternary gradient pump coupled with a 0.32 × 100 mm BioBasic strong cation exchange column). A flow rate of 3 μL/min was used for both SCX and RP columns.

A salt gradient was applied in steps of 0, 10, 15, 20, 25, 30, 35, 40, 45, 50, 57, 64, 90, and 700 mM ammonium acetate in 5% ACN, 0.1% formic acid, and the resultant peptides were loaded directly into the sample loop of a 0.18 × 100 mm BioBasic C18 reverse phase liquid chromatography column of a Proteome X workstation (ThermoElectron). The reverse phase gradient used 0.1% formic acid in ACN and increased the ACN concentration in a linear gradient from 5% to 30% in 20 min and then 30% to 95% in 7 min, followed by 5% for 10 min for 0, 10, 15, 25, 30, 45, 64, 90, and 700 mM salt gradient steps. For 20, 35, 40, 50 and 57 mM salt gradient steps ACN concentration was increased in a linear gradient from 5% to 40% in 65 min 95% for 15 min and 5% for 20 min.

The mass spectrometer was configured to optimize the duty cycle length with the quality of data acquired by alternating between a single full MS scan followed by three tandem MS scans on the three most intense precursor masses (as determined by Xcalibur software in real time) from the full scan. The collision energy was normalized to 35%. Dynamic mass exclusion windows were set at 2 min, and all of the spectra were measured with an overall mass/charge (m/z) ratio range of 300–1700.

All searches were done using TurboSEQUEST™ (Bioworks Browser 3.2; ThermoElectron). Mass spectra and tandem mass spectra were searched against an *in silico *trypsin-digested database of bovine RefSeq proteins downloaded from the National Center for Biotechnology Institute [NCBI; 12/26/2006; 24,853 entries]. Trypsin digestion including mass changes due to cysteine carbamidomethylation (C, 57.02 Da) and methionine mono- and di-oxidation (15.99 Da and 32 Da), was included in the search criteria. The peptide (MS precursor ion) mass tolerance was set to 1.5 Da and the fragment ion (MS^2^) mass tolerance was set to 1.0 Da. Rsp Value less than 5.

As a primary filter we first limited our Sequest search output to include only peptides ≥ 6 amino acids long, with ΔCn ≥ 0.08 and Sequest cross correlation (Xcorr) scores of 1.5, 2.0 and 2.5 for +1, +2, and +3 charge states, respectively. We next used a decoy database search strategy [49] (using the same primary filter for the real database search) to calculate P values for peptide identifications as this allows us to assign the probability of a false identification based on the real data from the experiment itself [49-52]. Since the accuracy of peptide identification depends on the charge state we calculated P values for +1, +2, and +3 charge states separately. The probability that peptide identification from the original database is really a random match (P value) is estimated based on the probability that a match against the decoy database will achieve the same Xcorr [51,53]. Protein probabilities were calculated exactly as described [54,55] using only peptides with a P < 0.05 and only those proteins were used for further modeling. All protein identifications and their associated MS data have been submitted to the PRoteomics IDEntifications database (PRIDE ;[56]) and PRIDE accession numbers are 1883–1888.

### Differential protein expression

Label free quantification approaches design to quantify relative protein abundances directly from high throughput proteomic analyses with out labeling techniques. Here, we used *ProtQuant *[29], a java based tool for label free quantification that uses a spectral counting method with increased specificity (and thus decreased false positive i.e. type I errors). This increased specificity is achieved by incorporating the quantitative aspects of the Sequest cross correlation (XCorr) into the spectral counting method. *ProtQuant *also computes the statistical significance of differential expression of control and treatment for each protein using one-way ANOVA (α ≤ 0.05). This method requires at least 3 peptides for each protein from the combination of the control and treatment before to calculate a p-value.

### Gene Ontology Annotation

We used Gene Ontology (GO) resources and tools available at AgBase [57] to identify the molecular functions and biological processes represented in differentially expressed proteins in our datasets. We used *GORetriver *tool to obtain all existing GO annotations available for known proteins in our datasets. We first GO-annotated differentially expressed proteins in our datasets using existing annotations from probable orthologs with ≥90% sequence identity using the UniRef 90 database. Proteins without annotation at UniRef 90, but between 70–90% sequence identities to presumptive orthologs with GO annotation were GO-annotated using *GOanna tool *[22]. Biological process annotations for these proteins were grouped into more generalized categories using *GOSlim viewer *[22].

### Modeling using Ingenuity pathway analysis

To gain insights into the biological pathways and networks that are significantly represented in our proteomic datasets we used Ingenuity Pathways Analysis (IPA; Ingenuity Systems, California). Currently IPA accepts gene/protein accession numbers from human, mouse, and rats only. Therefore, to use IPA, we mapped bovine proteins from our datasets to their corresponding human orthologs by identifying reciprocal-best-BLAST hits and uploaded these accession numbers into IPA. IPA selects "focus genes" to be used for generating biological networks. Focus genes are based on proteins from our datasets that are mapped to corresponding gene objects in the Ingenuity Pathways Knowledgebase (IPKB) and are known to interact with other genes based on published, peer reviewed content in the IPKB. Based on these interactions IPA builds networks with a size of no more than 35 genes or proteins. A P-value for each network and canonical pathway is calculated according to the fit of the user's set of significant genes/proteins. IPA computes a score for each network from P-value and indicates the likelihood of the focus genes in a network being found together due to chance. We selected networks scoring ≥ 2, which have > 99% confidence of not being generated by chance [58,59].

Biological functions are assigned to each network by using annotations from scientific literature and stored in the IPKB. Fisher exact test is used to calculate the P-value determining the probability of each biological function/disease or pathway being assigned by chance. We used P ≤ 0.05 to select highly significant biological functions and pathways represented in our proteomic datasets [58].

## Authors' contributions

DP performed the proteomics sample preparation, data generation, analyzed and interpreted proteomic data, systems biology modeling and analysis and wrote the draft of the manuscript. BN developed the biomarker discovery computational tools, participated in design of this study and helped to interpret the systems biology modeling. AK and JF did sample collection and pre-proteomic sample preparation. EM facilitated sample collection, contributed to design of the study, provided expert knowledge and interpretation in reproductive biology and helped to draft the manuscript. SCB conceived of the study, participated in its design and coordination, helped analyze and interpret the statistical analysis of the proteomics data and helped to draft the manuscript. All authors read and approved the final manuscript.

## Supplementary Material

Additional file 1 Proteins identified by DDF-MudPIT and their distribution in high or low fertility group spermatozoa. Column A show the GI numbers of the identified proteins, Column B indicates the corresponding protein names (assigned by NCBI). Column C shows the protein distribution in high or low fertility group spermatozoa or common to both (HF: High fertility group spermatozoa; LF: Low fertility group spermatozoa; C: common to both). For each protein we provided the information about number of peptides, Sequest cross correlation score (∑Xcorr) and DDF fraction information (DDF1, 2, 3 and 4). DDF sequentially extracts proteins from different sub cellular locations. DDF1, 2, 3, 4 corresponds to cytosolic, membrane, nuclear and cytoskeletal fractions respectively [18, 60]. We identified few proteins in more than one DDF fraction. This may be due to membrane proteins identified in all DDF fractions with increasing number of transmembrane domains in each DDF fraction. Many of the proteins that function in the nucleus at some stage may be present in the cytoplasm and can thus be found in all the fractions [18]. Click here for file

Additional file 2 Fertility data of bulls whose sperm samples were used for this study. For each bull we provided the information about bull number, number of services, percent difference from average breeding rate and standard deviation. Sperm samples from three high fertility (HF) bulls were pooled as HF group, and Sperm from three low fertility (LF) were pooled as LF group. Click here for file
